# Mechanical Analysis of Ceramic/Polymer Composite with Mesh-Type Lightweight Design Using Binder-Jet 3D Printing

**DOI:** 10.3390/ma11101941

**Published:** 2018-10-11

**Authors:** Dong-Hyun Kim, Jinwoo Lee, Jinju Bae, Sungbum Park, Jihwan Choi, Jeong Hun Lee, Eoksoo Kim

**Affiliations:** 3D Printing Manufacturing Process Center, Korea Institute of Industrial Technology (KITECH), Ulsan 44413, Korea; zenith1179@kitech.re.kr (J.L.); bjj00704@kitech.re.kr (J.B.); sbred@kitech.re.kr (S.P.); choijh@kitech.re.kr (J.C.); pocion@kitech.re.kr (J.H.L.); osgim@kitech.re.kr (E.K.)

**Keywords:** binder jet, 3D printing, light weight structure, sand mold, ceramic/polymer composite

## Abstract

3D printing technology has recently been highlighted as an innovative manufacturing process. Among various 3D printing methods, binder jetting (BJ) 3D printing is particularly known as technology used to produce the complex sand mold quickly for a casting process. However, high manufacturing costs, due to its expensive materials, need to be lowered for more industrial applications of 3D printing. In this study, we investigated mechanical properties of sand molds with a lightweight structure for low material consumption and short process time. Our stress analysis using a computational approach, revealed a structural weak point in the mesh-type lightweight design applied to the 3D-printed ceramic/polymer composite.

## 1. Introduction

A casting process has played an important role in the field of conventional metal technology for thousands of years. As a manufacturing method for various machines, automobiles, ships and their parts, the casting process is still being selected with a high portion: Machine tools (81%), electrical machinery (80.8%), textile machinery (50%), ship machinery (99.2%), and automobiles (13%) [1]. Nowadays, a primary paradigm shift is however occurring from the classical mass production to smaller and customized amounts. This change in manufacturing environment is requiring a faster and more flexible production process such as additive manufacturing.

The additive manufacturing technology called 3D Printing is attracting attention as one of the representative technologies that can lead the 4th Industrial Revolution [2]. In fact, 3D printers are actively applied to various fields of electronics [3], telecommunications [4], biology [5], and industrial construction [6]. Moreover, 3D printed modules and frameworks are used also in many medical fields, such as cardiothoracic surgery [7], orthopedics [8], neurosurgery [9] and dentistry [10]. Actually, 3D printers can be used alone or in combination with 3D radiologic [11] or optical scansion devices [12]. The International Organization for Standardization (ISO)/American Society for Testing and Materials (ASTM) have classified 3D printing technology based on lamination methods into seven categories: Photo polymerization (PP), material extrusion (ME), binder jetting (BJ), material jetting (MJ), direct energy deposition (DED), powder bed fusion (PBF), and sheet lamination (SL) at ISO/ASTM 52900:2015 [13].

Among the various 3D printing techniques, binder-jetting is a method used to bind together powder particles by selectively jetting a polymer binder layer-by-layer to form a green part. Particularly, a sand BJ 3D printer has a large benefit as it makes complicated sand molds directly without a wooden mold, which is necessary in a conventional sand mold process. It is also possible to produce sand molds using BJ 3D printing, which is faster than using the conventional casting method. For example, BJ 3D printing quickly makes sand molds with integrated gating systems, embedded cores, and without the need for a wooden mold [14]. However, the high material and process costs of BJ 3D printing need to be lower for a wide industrial application of 3D printing.

Previous studies [15,16] have focused on evaluating characteristics of the sand molds manufactured by 3D printing in comparison with the conventional method, and on reducing the material consumption in 3D-printed molds with a shell structure. Snelling et al. [17] have adopted BJ 3D printing to produce sand molds with a complex pattern. In a study by Shangguan et al. [18], they fabricated molds with a shell-truss structure using 3D printing, reducing the use of materials by two-thirds compared to conventionally-made molds. In addition, the cooling time of melting aluminum could be successfully controlled by the application of lightweight structures [19]. Deng et al. [20]. who also suggested a new lightweight design of sand molds with the air cavity on riser using a BJ 3D printer, found that solidification time can improve by 12.5% through an insulation effect at the riser cavity.

As mentioned above, only macroscopic designs have been previously studied for the additive manufacturing of sand molds. The macroscopic design for reducing the volume of sand molds depends on their shape. However, a lightweight design with a smaller length scale level, such as a pattern, may be possibly applied regardless of the shape of sand molds, and thus will considerably increase the adaptability of Design for Additive Manufacturing (DfAM) in BJ 3D printing.

In this work, we try to find a methodology of the lightweight design in a smaller length scale for BJ 3D printing, such as a typical conformal lattice cell in metal. To investigate the mechanical properties of lightweight designs, we introduce a basic unit block sample of a ceramic/binder composite applied to a whole sand mold using a BJ 3D printer in this study. The selection of two different structures was just done in this research for the purpose of comparing a typical lightweight design for metal with our ideal structure. We also address geometrical effects, such as the size and shape of typical lightweight patterns provided by commercial DfAM software on the basis of mechanical property evolution.

## 2. Experimental and Simulation Set-Up

**Figure 1:**
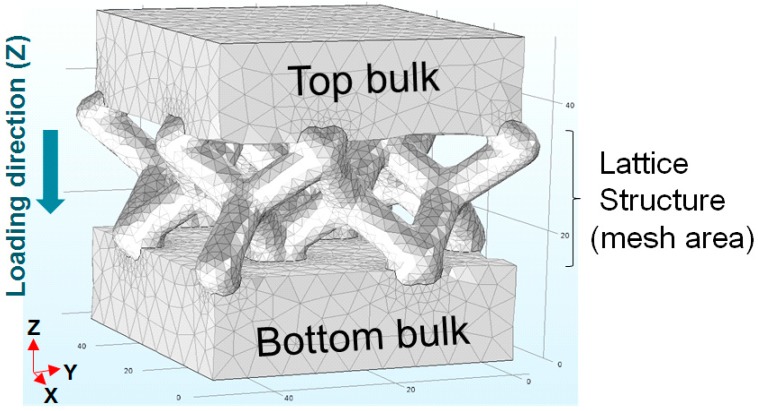
The image of a mesh-type sample having a lattice structure and solid bulk pads at the top and bottom.

To examine basic design factors of a lightweight structure for a sand/polymer composite, two types of lightweight structures were introduced in this work: a box with square holes (Type-1) and a lattice with upper and bottom pads (Type-2). Type-2 was designed with typical patterns for metals using a commercial topology optimization software (3-matic, ver.12, Materials company in Leuven, Belgium) as shown in Figure 1. Both types of the sample have a cubic dimension of 50 mm. In the mesh type sample, the size of the mesh relatively decreases while the height of the bulk of the top and bottom increases to 6 mm, 8 mm, 10 mm, and 15 mm, and the total height of mesh type sample is fixed to 50 mm.

The lightweight specimens were printed using VSX1000 model (Voxeljet company in Friedberg, Germany), sand BJ 3D printer. For sand BJ 3D printing we adopted the sand (GS19, Strobel Quarzsand company in Freihung, Germany) with a medium grain size of 0.198 mm, and the binder (VX-2C Type B, Voxeljet company in Freiberg, Germany). The thickness of the sand layer was about 300 μm during the printing process. The binder-type was furan resin, which chemically reacted as cold hardening with the activator coated at sand surface.

The compression test which was based on a conventional method, following the procedures of Korean Standard (KS A 5301:1995) and the American Foundry Society [21] has been modified by placing samples between circular steel plates [22,23,24] in this work. Our lightweight-designed samples are evaluated using a universal testing machine (KDMT-156, Kyung Do Precision Co., Ltd., in Kyungi-do, Republic of Korea). The compression test was carried out at a rate of 1.2 mm/min under a load of 10 tons [25,26,27]. Each test was conducted twice for data accuracy. The test samples were broken by initiation of cracks, and no creep behavior was observed during testing.

We also conducted computational analysis with the conditions shown in Table 1, in order to predict stress distribution and fracture under uniaxial-loading. To reveal the correlation of lightweight degree and strength, the volume ratio (ρ/ρ_0_) is particularly defined as the lightweight-designed volume (ρ) divided by the initial cubic volume (ρ_0_) in this study. In this work, all graphs were plotted by Microsoft Excel software (Redmond, DC, USA). COMSOL Multiphysics software (ver.5.2, Burlington, MA, USA) also was used for computational analysis. No data analysis has been performed as each test was conducted twice for data accuracy. Tests have been conducted only two times as the difference between the two measures has been recorded to be very low.

## 3. Results and Discussion

### 3.1. Lightweight Designs and Strength

To obtain an optimum lightweight pattern design for a ceramic/polymer composite using the BJ 3D printing technique, it is necessary to consider two competing factors: Structural strength and ease of taking out unbound sand powders. Normally, lightweight designs decrease the total weight by regularly arranging an empty space inside a structure. While an excessive hollow volume inside the ceramic/polymer composite results in severe strength drop, a low unfilled one can prevent the unbound sand particles from getting out from the inside of vacant units perfectly. It is thus significant to address the relation between strength and hollow volume density considering the taking-out of unbound sand powders.

**Figure 2:**
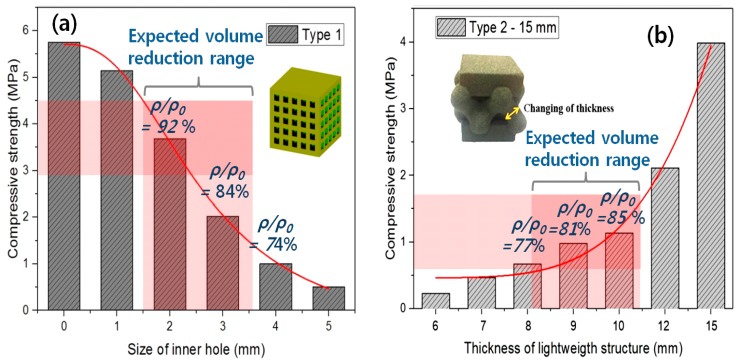
Change of compressive strength (σ_C_) by (**a**)-hole size and (**b**)-lattice beam thickness at two different types of samples: Type-1—cube with square holes, Type-2—mesh structure with pads. The definition of volume ratio (ρ/ρ_0_) was previously mentioned in the chapter of ‘experimental and simulation set-up’. In Type-2, each sample according to its pad thickness is called T (e.g., 12 mm →12 T).

For the purpose of controlling the hollow volume density in this work, we introduce two types of geometrical unit patterns: reducing total volume by arraying square holes and increasing the total one by increasing lattice beam thickness (see small sample images of Figure 2). The compressive strength (σ_C_) curves of two lightweight designs are shown in Figure 2.

Figure 2a is a graph of compressive strength (σ_C_) of the sand mold specimen with square holes as a function of size of the square holes. In the case of the specimen with 1 mm inner hole, it is difficult to remove the inner unbound sand powders. It turns out the size of the inner hole should be at least 2 mm to take out the inner sand powders clearly. σ_C_ and ρ/ρ_0_ decrease as the square hole size increases. A bulk volume with no holes indicates that the inner hole size is zero in Figure 2a. The σ_C_ value, ~5.7 MPa, of the bulk volume decreases to ~20% of the initial one, with the hole size of 4 mm corresponding to a ρ/ρ_0_ of 74%.

**Figure 3:**
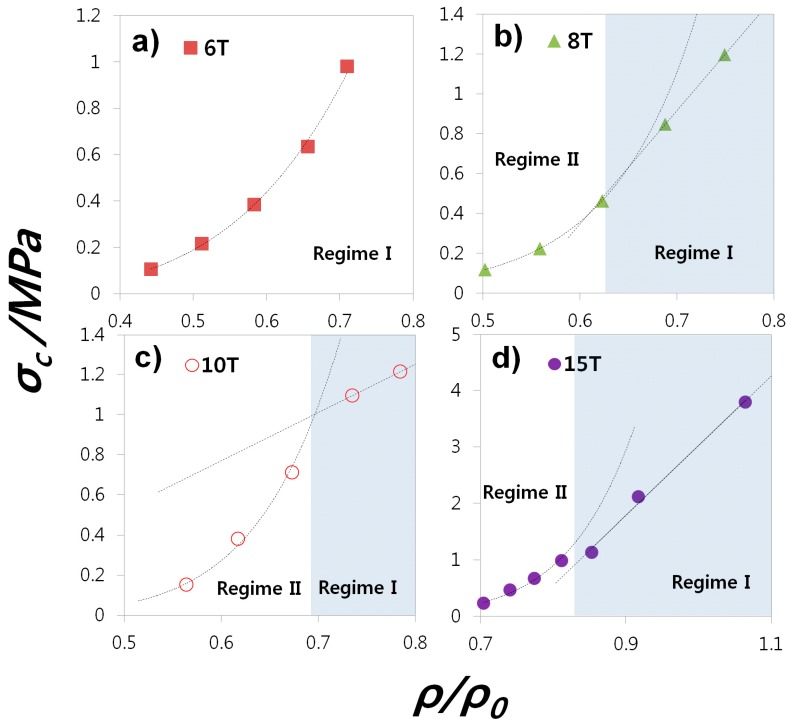
Various compressive strength (σ_C_) plot as a function of volume ratio (ρ/ρ_0_) at different pad (bulk) thickness (***t***_pad_ = (**a**) 6 T, (**b**) 8 T, (**c**) 10 T and (**d**) 15 T).

Figure 3b shows the σ_C_ with a change in the lattice beam thickness in Type-2. To avoid an abnormal and easy fracture at the top and bottom of lattice structures interfacing with a tester during a uniaxial compression test, a solid volume, acting as a pad, with a 15-mm thickness (15T) is added to the top and bottom faces of the samples, respectively [28,29]. Basically, σ_C_ increases as the lattice beam thickness increases. Additionally, σ_C_ values increase more largely in high density samples (12T and 15T of Figure 2b) with a lattice-type (Type-2), compared with those of the hole-type samples (Type-1) in Figure 2a. Adopting a thin lattice beam thickness normally applied to a metal lightweight design on our sand mold structure, some samples with thick lattice thickness have an exaggerated mesh shape (see the sample image of Figure 2b) which exceeds the boundary of the original bulk volume. This may cause a large increase in the σ_C_ of thick lattice samples (12~15 mm) (Figure 2b). Although Type-1 has higher strength, it is more difficult than Type-2, to take-out sand particles from samples. Hence, our further studies will focus on enhancing the low strength of Type-2.

### 3.2. Design Factors of Mesh-Type Lightweight Structure

Dependence on the strength of our lightweight structures for the thickness of mesh beams and pads as design factors is investigated in this chapter. Figure 3 shows a change of σ_C_ by ρ/ρ_0_. In the case of pad thickness > 8 (8 T, 10 T, and 15 T), the relation between σ_C_ and ρ/ρ_0_ appears to be linearly proportional and exponential at high and low ρ/ρ_0_, respectively. As the pad size of our mesh-type structure decreases, a transition from dual (Regime-II) to single (Regime-I) correlation takes place. The single plot of σ_C_-ρ/ρ_0_ at whole ρ/ρ_0_ range is a reasonable mechanical response of a pure mesh-structure. The exponential curve of 6 T shown in Figure 3a is thus considered typical behavior of the mesh-type ceramic/polymer composite. On the other hand, the linear plot of σ_C_-ρ/ρ_0_ which appears from Figure 3b–d seems to be a mechanical response of bulk rather than mesh since observed with a thick pad thickness (>8 T) and a high ρ/ρ_0_ (Regime II). This behavior is defined as ‘pseudo-bulk’ in this work. The pseudo-bulk is not real solid bulk, but it is still a mesh-type bulk. It is suggested that its different mechanical response, less sensitive strength drop with decreasing ρ/ρ_0_, may be caused because the local stress concentration, which results in a fracture, is lower than in mesh-type structures with low ρ/ρ_0_. Thin mesh beam structures are, hence, more fragile.

**Figure 4:**
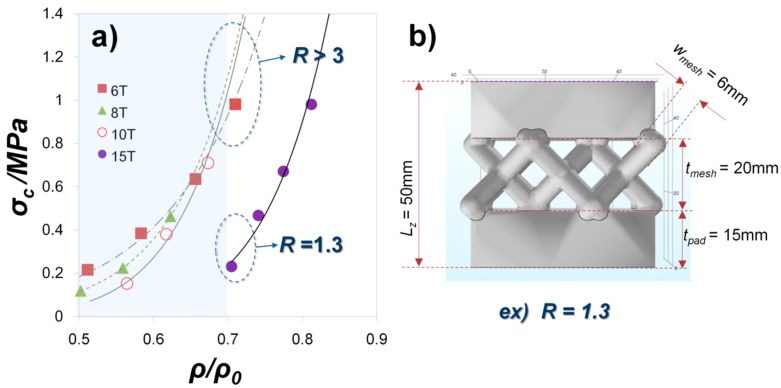
(**a**)-Compressive strength (σ_C_) as a function of volume ratio (ρ/ρ_0_) with different pad (bulk) thickness (***t***_pad_ = 6 T, 8 T, 10 T and 15 T) and (**b**)-thickness ratio of mesh and bulk (R = ***t***_mesh_/***t***_pad_).

We also examine compressive strength by a relative portion of pad and mesh at our mesh-type sample as shown in Figure 4a. The mesh-type sample has a vertical length (***l***_z_) of 50 mm in Figure 4b. Since the samples consist of top and bottom mesh and pads, the thickness of both pad (***t***_pad_) and mesh (***t***_mesh_) portions can vary in relation to 2 × ***t***_pad_ + ***t***_mesh_ = ***l***_z_. The pad thickness is changed from 6 mm to 15 mm, which results in a different pad-mesh ratio (R = ***t***_mesh_/***t***_pad_) and volume ratio (ρ/ρ_0_). As ρ/ρ_0_ decreases by applying the lightweight mesh-type structure, σ_C_ gradually decreases in all samples with a pad thickness of 6 mm, 8 mm, 10 mm, and 15 mm (6 T, 8 T, 10 T, and 15 T). When ρ/ρ_0_ is close to 0.7, the σ_C_ value at 15 T is much lower than those at 6 T, 8 T and 10 T. Whereas the R value of 15 T sample is 1.3 at ρ/ρ_0_ = 0.7, all other samples which show higher σ_C_ values satisfy ‘R > 3’. Therefore, it turns out that thick pads in a mesh-type lightweight structure seriously decrease in strength. The present report is very unique in its study of the relation between mechanical analysis and structural design in the ceramic 3D printing as no other studies have evaluated this specific topic.

### 3.3. FEM Analysis

**Figure 5:**
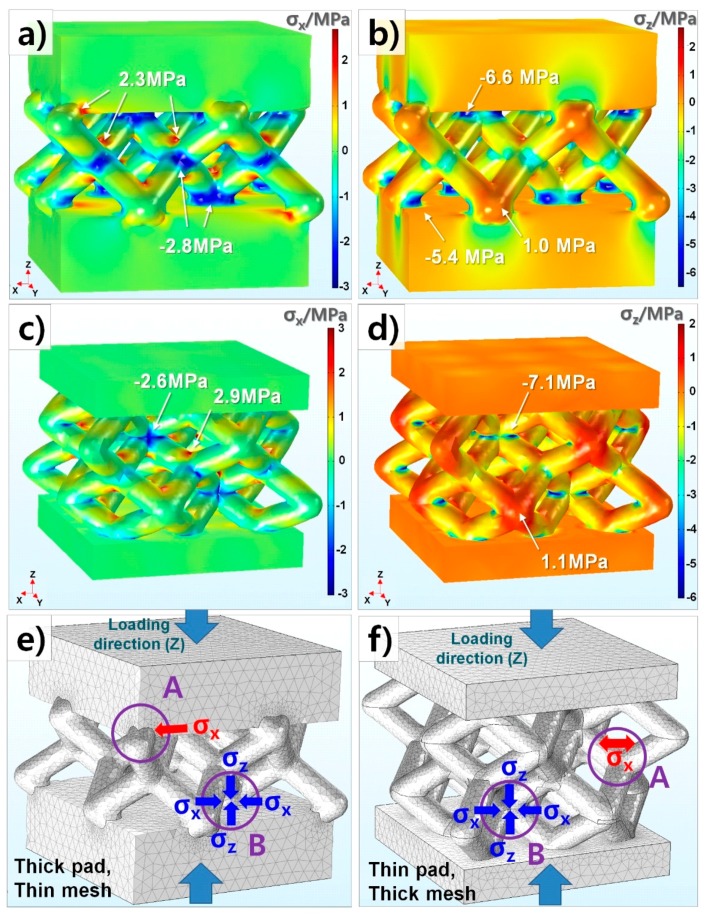
Mechanical stress analysis of lightweight designs with 15T-6 mm and 8T-10 mm (pad thickness and mesh beam width). (**a**) σ_x_ distribution (x-direction stress) of 15T-6 mm (**b**) σ_z_ distribution (z-direction stress) of 15T-6 mm (**c**) σ_x_ distribution of 8T-10 mm (**d**) σ_z_ distribution of 8T-10 mm (**e**) Stress concentration of 15T-6 mm (***t***_pad_ > ***t***_mesh_), A and B: Tensile and compressive stress at the interface of the mesh and pads, respectively (**f**) Stress concentration of 8T-10 mm (***t***_pad_ < ***t***_mesh_), A and B: Tensile and compressive stresses at internal mesh structure, respectively.

To analyze the effect of structural factors on mechanical fracture in detail, FEM simulation is introduced in this study. In view of stress to x and z-direction (σ_x_ and σ_z_), the mesh-type sample with ***t***_pad_ = 15 mm and ***t***_mesh_ = 6 mm which was seen to be particularly low σ_C_ (0.23 MPa) at ρ/ρ_0_ = 0.7 (Figure 4) is compared with that of ***t***_pad_ = 8 mm and ***t***_mesh_ = 10 mm (σ_C_ = 1.19 MPa), as depicted in Figure 5. Figure 5 illustrates the mechanical stress distribution of two samples with 15T-6 mm (***t***_pad-mesh_) (Figure 5a,b) with 8 T-10 mm (***t***_pad-_***t***_mesh_) (Figure 5c,d). Since our mesh samples show 4-fold symmetry at the z-axis, σ_x_ and σ_y_ should be almost the same. σ_x_ distribution is thus seen without σ_y_. The stress concentration mainly occurs at the mesh areas of both samples. As observed in Figure 5a–d, the samples 15 T-6 mm and 8 T-10 mm do not show any big stress differences which enable much lower σ_C_ of the former. While the sample with 15 T-6 mm gets higher tensile and compressive stress concentration (A,B) at the mesh and pads interface in Figure 5e, the one with 8 T-10 mm does at internal mesh structure in Figure 5f. It is expected that easy cracks are initiated at a comparatively weak boundary between the mesh and pads of 15 T-6 mm.

## 4. Conclusions

In this work, we studied fundamental design factors of lightweight structures for a BJ 3D printer and carried out those mechanical evaluations using experiments and FEM simulations.

(1) Compressive strength was measured with two lightweight designs, a cube with square holes (Type-1), a mesh structure with pads (Type-2), and the strength of both which remarkably decrease with the increasing volume ratio (ρ/ρ_0_). It turns out that the size of the inner hole of the Type-1 sample should be at least 2 mm for taking out the inner sand powders clearly. Although Type-1 has higher strength, it is more difficult than Type-2 to take out sand particles from samples. Hence, our further study will focus on enhancing the low strength of Type-2.
(2) With mesh-type lightweight structures, increasing pad thickness and decreasing a mesh area results in increasing the local stress concentration at the interface of the mesh and pads. It is expected that easy crack is initiated at a comparatively weak boundary between mesh and pads in the case of thick pad thickness.
(3) Since a commercial software for topology optimization provides lightweight designs for rigid single component materials such metals or plastics, it is not suitable to apply the lightweight designs to a ceramic/polymer composite with different mechanical behaviors. As a result, new types of light weight structures for sand casting molds are required to spread BJ 3D printing technology to the foundry industry.
(4) Further study will suggest and evaluate the new lightweight and rigid design of for additive manufacturing of a ceramic/polymer composite. It will reveal the correlation between structural and mechanical factors of the lightweight designs in detail.

## Figures and Tables

**Table 1:** Analytical methods and conditions used in computer numerical analysis.

Software	Compression with Prescribed Velocity	Loading Direction	Compression Time	Mechanical Analysis
COMSOL Multiphysics^®^	−2 × 10^−5^ m/s	Z-direction	0.1 s	Using principal stress (σ_XX_, σ_YY_, σ_ZZ_)
